# Evaluation of Maxillary Sinus Membrane Morphology Using a Novel Hybrid CNN-ViT-Based Deep Learning Model: An Automated Classification Study

**DOI:** 10.3390/diagnostics16050777

**Published:** 2026-03-05

**Authors:** Nurullah Duger, Furkan Talo, Gulucag Giray Tekin, Burak Dagtekin, Mucahit Karaduman, Muhammed Yildirim, Tuba Talo Yildirim

**Affiliations:** 1 Department of Periodontology, Faculty of Dentistry, Firat University, Elazig 23119, Turkey; bdagtekin@firat.edu.tr (B.D.);; 2 Digital Transformation and Software Office, Rectorate, Firat University, Elazig 23119, Turkey; ftalo@firat.edu.tr; 3 Department of Periodontology, Faculty of Dentistry, Batman University, Batman 72060, Turkey; 4 Department of Software Engineering, Malatya Turgut Ozal University, Malatya 44000, Turkey; mucahit.karaduman@ozal.edu.tr; 5 Department of Artificial Intelligence and Data Engineering, Firat University, Elazig 23119, Turkey; muhammedyildirim@firat.edu.tr

**Keywords:** artificial intelligence, deep learning, CNN, ViT, maxillary sinus, cone-beam computed tomography

## Abstract

**Objectives**: This study aimed to develop and validate a hybrid deep learning model combining Convolutional Neural Networks (CNN) and Vision Transformers (ViT) to automatically classify maxillary sinus membrane morphologies on Cone-Beam Computed Tomography (CBCT) images, distinguishing between Normal, Flat, Polypoid, and Obstruction types. **Methods**: A dataset of 959 CBCT images was collected and categorized into four morphological classes: Normal, Flat, Polypoid and Obstruction. A custom hybrid model was developed, integrating a lightweight residual CNN for local feature extraction, learnable weighted feature fusion with a bidirectional feature pyramid network and a Transformer encoder for global context modeling. The performance of proposed model was compared against six different architectures, including ResNet50, MobileNetV3L and standard ViT models, using accuracy, precision, recall and F1-score metrics. **Results**: The proposed hybrid model achieved the highest overall accuracy of 98.44%, outperforming six strong CNN and ViT models including ResNet50 (97.92%) and ViT-B16 (86.46%) models. In class-wise analysis, the model demonstrated superior diagnostic capability, particularly for the “Obstruction” class, achieving 100% accuracy. High discrimination was also observed for “Flat” (98.21%) and “Polypoid” (98.04%) morphologies, confirming the model’s sensitivity to shape-based features. **Conclusions**: The proposed hybrid CNN-ViT model successfully classifies maxillary sinus membrane morphologies with high accuracy, effectively overcoming the limitations of standard ViT models on limited datasets. Detection of membrane morphology is vital for predicting surgical risks like membrane perforation and post-operative sinusitis. This model serves as a reliable clinical decision support tool, enabling clinicians to objectively assess specific risk factors before implant surgery and sinus floor elevation.

## 1. Introduction

The maxillary sinus is an air-filled cavity located in the posterior region of the maxilla with a volume of approximately 12.5 mL. It is the largest of the paranasal sinuses and opens into the middle meatus of the nasal cavity. The structure is characterized by its pyramidal form [1,2]. The membrane of the maxillary sinus is covered by a vascularized pseudostratified ciliated epithelium. The sinus membrane’s average thickness varies between 0.8 and 1 mm. Its visibility is not apparent in radiographic examinations of a healthy sinus [3]. In cases of bone insufficiency in the posterior maxillary region, this membrane is critical in sinus lift procedures. Additionally, the sinus membrane has been found to contain stem cells that have the capacity to promote bone formation. However, beyond its anatomical characteristics, the precise morphological assessment of this membrane is a critical prerequisite for surgical safety. Accurate identification of pathological variations is essential to determine the feasibility of sinus floor elevation, prevent intraoperative membrane perforation, and ensure the long-term survival of dental implants [4].

A study documented a prevalence of maxillary sinus pathologies of 56.3% [5]. The most prevalent of these is membrane thickening, which was observed in between 25.1% and 66% of cases [6]. Sinus membranes with a thickness of 2 mm or greater are considered pathological and are visible on radiographs. Local or systemic factors that cause acute or chronic inflammation can increase membrane thickness 10–15-fold, resulting in different radiographic findings, such as peripheral or local (polypoid or irregular) filling of the entire sinus [7]. In particular, thickening resulting from chronic inflammation, loss of ciliated cells, an increase in goblet cells, and metaplastic changes can cause damage to the sinus epithelium, leading to loss of function. Functional loss can also impair sinus drainage, jeopardizing the success of sinus lift procedures performed prior to dental implant surgery due to potential functional loss caused by sinus membrane thickening [8].

In sinus lift operations performed for bone augmentation in implant surgeries, infection and an increased risk of complications can be caused by ostium occlusion or membrane perforation due to membrane thickness. This condition can cause ostium occlusion in 59.3% of patients with membrane thickness greater than 5 mm [4]. It has been stated that membrane thickness is related to the rate of sinus perforation and that the risk of perforation increases when the thickness is less than 0.5 mm or greater than 3 mm [9]. Therefore, detecting sinus membrane thickening with Cone Beam Computed Tomography (CBCT) before dental implant surgery is important for ensuring the operation’s success [10].

CBCT is a reliable imaging method that improves the accuracy of surgical planning and reduces complications when evaluating the maxillary sinus in three dimensions [11,12]. Although soft tissue contrast is limited, CBCT provides accurate and reproducible sinus mucosal thickness measurements. Furthermore, CBCT can reveal subtle anatomical changes that are not visible in two-dimensional images [13,14]. Consequently, CBCT is often employed in dental implant surgery for evaluating sinus anatomy, variations, and pathologies [15,16,17]. However, the visual interpretation of CBCT scans is subjective, time-consuming, and dependent on the clinician’s experience [18]. These limitations necessitate automated, objective tools. AI-based approaches can overcome these challenges by detecting subtle pixel-level patterns that are imperceptible to humans, thereby providing a standardized and consistent assessment of sinus pathologies regardless of the observer’s fatigue or experience level [19].

Artificial intelligence (AI) refers to systems that think and act like humans and can think and act logically [20]. This approach aims to mimic human intelligence and can extract meaningful representations from complex data structures, particularly through deep learning (DL) methods. Convolutional Neural Networks (CNN) constitute a significant subset of DL algorithms and can automatically learn basic and distinctive features in images. They have achieved high success rates in classification and detection problems using these features [21]. Thanks to these capabilities, CNN-based methods are used effectively in the analysis of complex medical images, such as CBCT images, and have produced promising results in various dental applications, including tooth numbering, periapical pathology detection, and mandibular canal identification [22,23,24]. Recent studies comparing the diagnostic performance of artificial intelligence models with human practitioners have demonstrated that AI systems can achieve accuracy levels comparable to or exceeding those of dental students and general dentists [25]. In a study assessing periodontal parameters, AI demonstrated higher consistency than general dentists and achieved diagnostic performance metrics similar to those of senior specialists, particularly in detecting attachment loss and alveolar bone loss [26]. Furthermore, AI-assisted interpretation has been shown to significantly enhance the diagnostic sensitivity and specificity of dental interns, effectively bridging the gap between novice and expert clinicians in identifying pathologies such as dental caries and periapical lesions [27]. In endodontic diagnostic tasks, AI models have consistently outperformed both junior and senior dental students, suggesting that these tools can serve as reliable, objective adjuncts in clinical training and decision-making [28]. However, most current studies are limited to binary classifications or anatomical descriptions in analyses related to the maxillary sinus region. The number of studies addressing different morphological types of maxillary sinus membrane thickening in a systematic, multi-class manner is limited. Furthermore, the absence of standardised, multi-class labelled open or original datasets in this field restricts the comparability and clinical validity of the developed methods.

This study aims to develop a hybrid DL model that can automatically detect and classify sinus membrane thickening morphologies, which is critical for bone augmentation in dental implant surgery, using a unique dataset. The proposed model consists of a lightweight residual CNN-based feature extraction structure, a multi-scale feature fusion mechanism based on BiFPN, and Transformer encoder components capable of learning global contextual relationships. The model’s performance has been thoroughly tested using quantitative metrics.

## 2. Materials and Methods

### 2.1. Acquisition of Images and Dataset

This retrospective study was approved by the ***Firat*** University Non-Interventional Ethics Committee (FUGOEK) (No. 2025-17-38) and conducted in accordance with the Declaration of Helsinki. A total of 1000 CBCT images obtained from patients who applied to the ***Firat*** University, Faculty of Dentistry between January 2019 and September 2025 were examined. CBCT images were obtained using a ProMax 3D Mid (Planmeca, Helsinki, Finland) tomography device with a standard imaging protocol of 90 kVp and 8 mA for an exposure time of 8–9 s. The voxel size of the images was 0.4 mm. The images were analyzed by two experts using Romexis Viewer 4.4.3 (Planmeca, Helsinki, Finland) software, according to the manufacturer’s recommendations, in a configuration window where cross-sections could be viewed at 0.2 mm intervals, directly from the raw images without any preprocessing.

**Figure 1:**
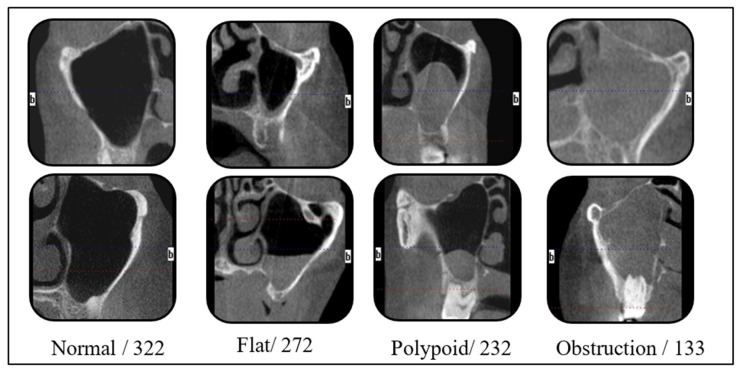
Sample images and numbers for each class.

A dataset consisting of 959 PNG format images was created, classified into four categories: Normal, Flat, Polypoid and Obstruction. All radiographic data used in this study were retrospectively obtained from patients, who had previously provided written informed consent for the anonymous use of their data for scientific purposes. This consent was obtained during the patient’s initial visit to the clinic. Therefore, no additional informed consent was required. Data were anonymized to prevent identification of patients. Our dataset, consisting of 4 classes and a total of 959 images created using CBCT cross-sectional images for the automatic detection of the sinus morphologies, is presented in Figure 1.

### 2.2. Manual Measurements and Standardization

The classification of maxillary sinus membrane shape was performed independently by two periodontists (N.D., T.T.Y.) with over ten years of experience in dental implant surgery. Eighty CBCT sections (Normal (*n* = 20), Flat (*n* = 20), Polypoid (*n* = 20) and Obstruction (*n* = 20)) were selected, and manual analysis was repeated two weeks later to determine inter-observer and intra-observer consistency. Images on which the two experts disagreed were excluded from the study.

### 2.3. Proposed Model

**Figure 2:**
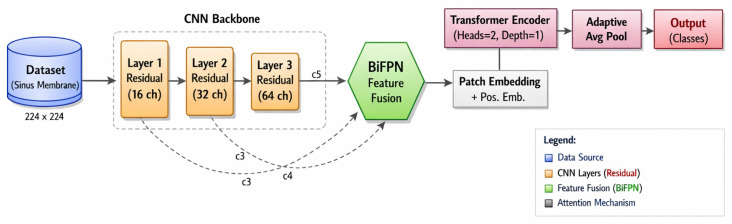
Flowchart of the proposed method.

This study develops an AI method capable of automatically detecting and classifying the thickening and shape of the maxillary sinus membrane, which is critically important for bone augmentation in dental implant surgeries. The proposed method effectively extracts local anatomical details from dental images, integrates information at different spatial resolutions, and models global relationships between membrane thickening patterns within the same architecture. For this purpose, multi-scale feature extraction is performed with a CNN with light residual connections, and the obtained features are combined with a BiFPN using learnable weights. Then, high-level feature representations are converted into a patch-based embedding structure and transferred to the ViT encoder layer, thus modeling long-range spatial dependencies. In the final stage, the obtained global feature representations are processed through the classification layer to generate a final prediction regarding the maxillary sinus membrane thickening status and shape. The end-to-end processing flow of the proposed method is presented in Figure 2.

As shown in the flowchart in Figure 2, the proposed method begins with feeding the input image into the model. Before being transferred to the network input, dental images are rescaled to a fixed spatial dimension and their density values are normalized to make them suitable for model training. This preprocessing step aims to reduce the effect of brightness and contrast variations that may arise from different imaging conditions and to ensure more stable model learning. After the preprocessing stage, the images are transferred to a CNN backbone containing lightened residual connections. In this stage, the network generates multi-scale feature maps representing the boundary structures of the maxillary sinus membrane, thickening regions, and local morphological features of the surrounding bone tissue at different spatial resolutions. Thanks to residual connections, information loss that may occur as depth increases is reduced, and low-level details are efficiently transferred to higher layers.

The resulting multi-scale feature maps are transferred to a Bidirectional Feature Pyramid Network (BiFPN) with learnable weights. At this stage, features at different resolution levels are combined both from top to bottom and bottom to top to create an enriched feature representation. The BiFPN structure contributes to a more distinctive representation of different thickening types by simultaneously considering the fine details of membrane thickening and the broader anatomical context [29,30].

Feature maps obtained from BiFPN are converted into vector arrays via a patch-based embedding mechanism. After this process, each spatially divided image segment is represented by a fixed-size marker vector. Following this step, spatial embedding is added to the marker array, incorporating spatial positional information. This allows for a more accurate modeling of the anatomical arrangement of the transformer-based structure within the image. The spatially enriched marker array is then transferred to the transformer encoder layer. Thanks to the self-attention mechanism, long-range spatial relationships between thickening regions in the maxillary sinus membrane are learned, and local findings are evaluated within a global context. This structure allows for more accurate differentiation of irregular and diffuse thickening patterns. In the final stage, the Transformer encoder outputs are summarized into a single representation vector through a global pooling process. This resulting global feature vector is transferred to the fully coupled classification layer, and the final class prediction regarding the maxillary sinus membrane thickening status and type is generated. The model is optimized during the training process using a multi-class cross-entropy loss function, and the network parameters that achieve the highest success on the validation data are saved as the final model. As shown in the flowchart in Figure 2, the method begins by feeding the input image into the model. Dental images are spatially rescaled and their density values are normalized before being transferred to the model. The input image is mathematically defined as $X\in \;R^{H\;\times \;W\;\times \;C}$, and the normalized input equation is given in Equation (1).

(1) $$X_{n}=\frac{X-\mu }{\sigma }$$

Here, *X* represents the raw image tensor representing the dental image, *H* and *W* represent the spatial dimensions of the image, *C* is the number of canals, $X_{n}$ is the normalized input image, μ is the average of the image intensity values, and σ is the standard deviation of the image intensity values.

Normalization reduces the effect of intensity variations across different imaging conditions, allowing the network to be trained on a more balanced input distribution. The normalized input image is fed into the CNN backbone, which includes a few residual connections. At this stage, local features are extracted using convolution, and boundary structures and thickening regions of the maxillary sinus membrane are highlighted. The output of a convolution layer is calculated as shown in Equation (2).

(2) $$F=W*X_{n}+b$$

In Equation (2), *F* represents the feature map obtained as a result of the convolution operation, W represents the learnable kernel weights of the convolution layer, and *b* is the bias term of the convolution layer. Thanks to the residual structure, information loss is reduced by directly adding the input information to the output. The residual block output is calculated as in Equation (3).

(3) $$Y=ReLU\left(F\left(X_{n}\right)+S\left(X_{n}\right)\right)$$

In Equation (3), Y represents the residual block output, $F\left(X_{n}\right)$ represents the main transformation within the residual block, $S\left(X_{n}\right)$ represents the shortcut link in the residual block, and ReLU is the nonlinear activation function. The process in Equation (4) results in multi-scale feature maps at different resolution levels, denoted as C3, C4, and C5. These maps are computed from the layers of the CNN architecture, as shown in Equation (4).

(4) $$\left(C_{3},C_{4},C_{5}\right)=f_{CNN}\left(X_{n}\right)$$

In Equation (4), $C_{3}$ represents the feature map obtained from the early layers of the CNN backbone, $C_{4}$ represents the feature map obtained from the mid-level layers, $C_{5}$ represents the feature map obtained from the deeper layers of the CNN backbone, and $f_{CNN}$ represents the CNN backbone with light residual connections. The multi-scale features obtained from Equation (4) are transferred to a BiFPN with learnable weights. In this stage, the channel sizes of the features across different resolutions are first equalized, then weighted and combined. These operations are expressed as in Equation (5).

(5) $$C_{i}^{\prime }=\phi_{1\times 1}\left(C_{i}\right),\;i\;\in \;\left\{3,\;4,\;5\right\}$$

In Equation (5), $C_{i}$ represents the feature map obtained from the CNN backbone, $C_{i}^{\prime }$ represents the transformed feature map obtained after the channel equalization process, and $\phi_{1\times 1}$ represents the 1 × 1-dimensional convolution operation. After the feature maps with adjusted dimensions and consistent properties are created, BiFPN operations are performed. The learnable weights are normalized as shown in Equation (6) to ensure numerical stability.

(6) $$\alpha_{k}=\frac{ReLU\left(w_{k}\right)}{\sum_{j}ReLU\left(w_{j}\right)+\varepsilon }$$

In Equation (6), $\alpha_{k}$ represents the normalized weighting coefficient used in the BiFPN structure, $w_{k}$ represents the learnable BiFPN weighting parameter, ε is a tiny positive constant added to the denominator to ensure numerical stability, and j is the number of all weights involved in the coupling process. Using these weights, multi-scale features are combined to obtain top-down enriched representations via Equations (7) and (8).

(7) $$P_{4}=\alpha_{1}C_{4}^{\prime }+\alpha_{2}↑\;\left(C_{5}^{\prime }\right)$$

(8) $$P_{3}=\alpha_{1}C_{3}^{\prime }+\alpha_{2}↑\;\left(P_{4}\right)$$

Here, $P_{4}$ represents the fourth-level intermediate feature map obtained as a result of top-down feature fusion in the BiFPN structure, $P_{3}$ represents the third-level feature map, $C_{3}^{\prime },\;C_{4}^{\prime },\;C_{5}^{\prime }$, represent CNN feature maps of different resolution levels to which the equalization process has been applied, ↑ represents the upsampling process, and $\alpha_{1},\;\alpha_{2}$ represent the normalized learnable BiFPN weighting coefficients.

Following the top-down feature-merging process in the BiFPN structure, an additional bottom-up fusion step is applied. At this stage, feature representations from different resolution levels are reassembled using learnable weights, and multi-scale information is integrated into a single coherent representation. The resulting top-level feature map contains both fine anatomical details and high-level contextual information and is used as input for the next stage.

This final feature map is converted into a patch-based embedding mechanism to be processed by Transformer-based architectures. In this process, the feature map is divided into fixed-size parts, and each part is computed using a token vector, as given in Equation (9).

(9) $$Z=Flatten\left(\phi_{N\times N}\left(P^{out}\right)\right)$$

Here, *Z* represents the token sequence obtained as a result of the Patch embedding operation, $P^{out}$ represents the final feature map of the BiFPN output, $\phi_{N\times N}\;$ represents the convolution operation with kernel size and step length *N × N*, Flatten(.) represents the operation that converts the spatial dimensions of the convolution output into a one-dimensional vector array, and *N* represents the patch size.

A learnable positional encoding representing spatial position is added to the token sequence obtained from the patch embedding process. The token sequence enriched with this positional information is then transferred to a Transformer encoder block with a self-attention mechanism. Here, long-range relationships between tokens are modeled, and thickening patterns in the maxillary sinus membrane are evaluated within a global context. The query (Q), key (K), and value (V) vectors are calculated as in Equation (10) [31].

(10) $$Q=ZW_{Q},\;K=ZW_{K},\;V=ZW_{V}$$

In Equation (10), *Z* represents the token sequence obtained by patch embedding and spatial information addition, *Q* represents the query vectors, *K* represents the key vectors, *V* represents the value vectors, and *W* represents the learnable weight matrices. The equation for the scaled attention mechanism is presented in Equation (11) [31].

(11) $$Att(Q,K,V)=softmax\left(\frac{QK^{T}}{\sqrt{d_{h}}}\;\right)V$$

When Equation (11) is examined, Att(Q,K,V) represents the output of the scaled dot-multiplication self-attention operation, $QK^{T}$ is the dot-multiplication matrix measuring the similarity between the query and key vectors, and $d_{h}$ represents the vector dimension for each heading used in the attention mechanism. The markers obtained from the transformer encoder layer are combined to form a feature representation that summarizes all spatial information. This process aims to combine local and global information about the maxillary sinus membrane into a single representation. In the next step, the resulting global feature representation is then sent to the classification layer. The classification layer uses the features learned by the developed model to generate class scores for the state and type of maxillary sinus membrane thickening. Thus, the model identifies the appropriate class for the test image. A multi-class cross-entropy loss function was used to obtain the difference between the predicted and actual values during the training of the developed model. The equation of the multi-class cross-entropy loss function is given in Equation (12).

(12) $$L_{CE}=-\sum_{c=1}^{C}y_{c}\log\left(p_{c}\right)$$

In Equation (12), $L_{CE}$ is the cross-entropy loss calculated for the inputs and labels, *C* is the number of classes, $y_{c}$ is the representation of the actual class label, and $p_{c}$ represents the class probability predicted by the model.

The performance of the proposed model and other models used in the study was evaluated using metrics such as accuracy, precision, sensitivity, and F1 score. Calculations are made using Equation (13) for Accuracy, Equation (14) for Precision, Equation (15) for Recall, and Equation (16) for F1-Score, which are performance metrics.

(13) $$\frac{TP+TN}{TP+TN+FP+FN}$$

(14) $$\frac{TP}{TP+FP}$$

(15) $$\frac{TP}{TP+FN}$$

(16) $$2\cdot \frac{\left(Precission\cdot Recall\right)}{\left(Precission+Recall\right)}$$

Here, TP represents a true positive, TN represents a true negative, FP represents a false positive, and FN represents a false negative.

To demonstrate the effectiveness of the proposed method, comparative analyses were conducted using different DL architectures commonly used in the literature during the experimental evaluation. Among the transformer-based models used in the study, Vision Transformer Base Patch16 (ViTB16) and Vision Transformer Base Patch32 (ViTB32) models [32] were selected. At the same time, among CNN-based approaches, the architectures DenseNet121 [33], EfficientNetB0 [29], ConvNeXtTiny [34], MobileNetV3Large [35], and ResNet50 [36] were selected. All comparison models were trained and evaluated on the same dataset split and with similar training settings, and the resulting results were comprehensively analyzed with respect to classification performance, generalization, and class-based discrimination of the proposed method.

### 2.4. Experimental Setup

The results obtained in this study were achieved using an NVIDIA Tesla T4 GPU in the Google Colab environment. 80% of the images in the dataset were used for training, while the remainder were used for testing. However, to monitor epoch-based performance, a 10% random subset of the training data was used for validation. The input image of the models is sized at 224 × 224. In the proposed model, a minibatch of 16 was used, AdamW was chosen as the optimizer, a learning rate of 5 × 10^−4^ was used, and the model was trained for 100 epochs. Multi-class cross-entropy was used as the loss function in the developed model. To compare the performance of the developed model, results were also obtained with current models frequently used in the literature. The pre-trained models used in our study were initialized with ImageNet weights. During training, model parameters were left unfrozen. The performance of these models was evaluated using different metrics.

## 3. Results

### 3.1. Validation of Manual Segmentation

The reliability of the analyses was assessed using intraclass correlation coefficients (ICCs) to evaluate inter-observer and intra-observer consistency. ICC values of 0.9451 and 0.9691 were observed for manual classification, respectively, indicating excellent intra-observer reliability. Furthermore, ICC values of 0.9525 and 0.9636 were also observed, demonstrating excellent inter-observer reliability. These results confirm the accuracy and consistency of the analysing process, as evidenced by the strong reliability of measurements taken by different operators and over time.

### 3.2. Experimental Results

**Figure 3:**
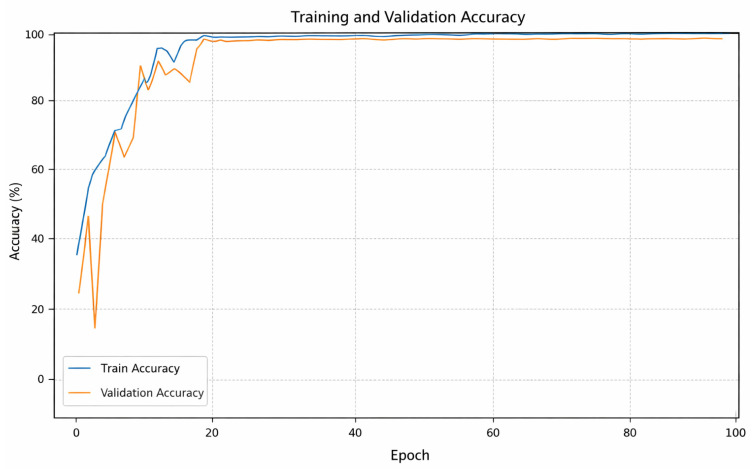
Proposed models’ accuracy curves.

This section presents the results of the proposed model and the pre-trained models used in the study for automatically classifying maxillary sinus membrane morphologies in CBCT. Examining the training and validation graph in Figure 3, we observe that the proposed model’s learning process forms a stable structure. A significant increase in training and validation success rates was observed in the first 20 epochs. After the first 20 epochs, the model completed the interval steadily at the upper point of the graph. The training and validation curves were very close, with a negligible difference between them. Interpreting these graph movements, it shows that the model does not have a problem memorizing the data and has a high generalization ability. After 100 epochs, both curves settled above the 98% band, indicating that the architecture had extracted meaningful information from the training data.

**Figure 4:**
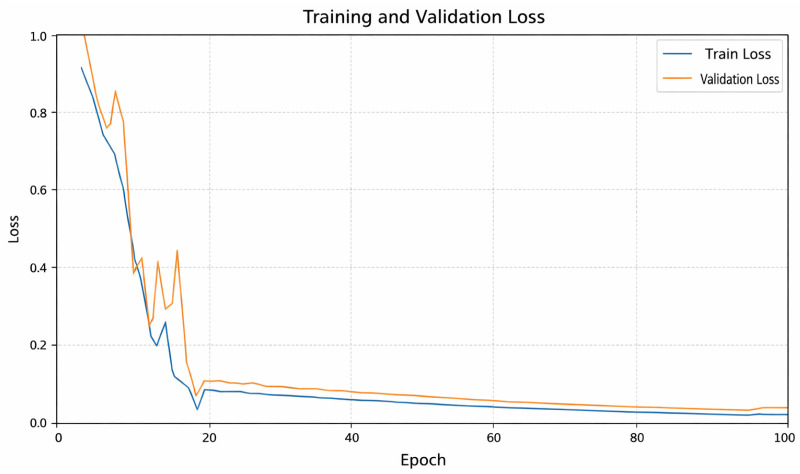
Proposed models loss curves.

The loss curve of the proposed model is presented in Figure 4. Figure 4 shows that the loss value decreases after the model training begins. After the 15th epoch, the model is seen to move away from local minima and towards a global minimum. After 20 epochs, the loss value falls below 0.1.

**Figure 5:**
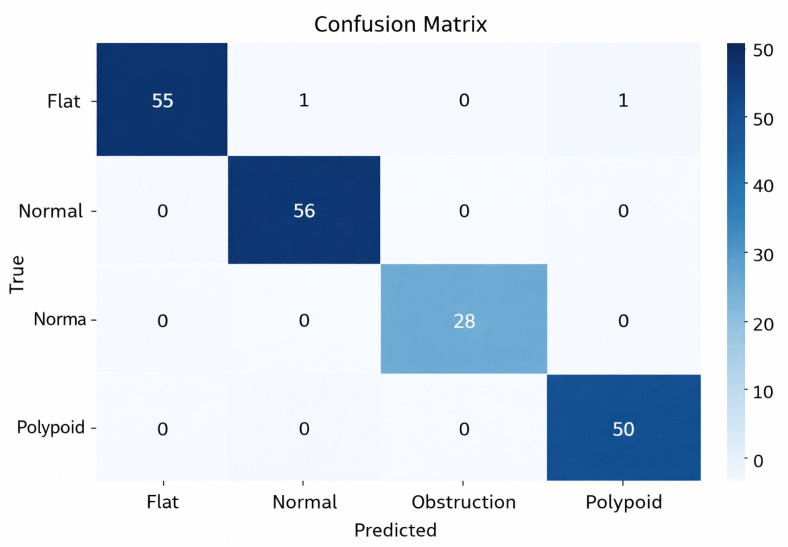
Proposed models confusion matrix.

The confusion matrix of the proposed model for automatically detecting maxillary sinus membrane thickening, which is critical for bone augmentation in dental implant surgeries, is presented in Figure 5. An examination of the confusion matrix for the proposed model shows that it performs exceptionally well, correctly predicting all 28 samples in the Obstruction class. It also correctly predicted all 56 test images in the Normal class, 55 out of 57 in the Flat class, and 50 out of 51 in the Polypoid class. When examining incorrect classifications, it was observed that errors occurred only between morphologically similar classes and were very few in number. This indicates that the model’s performance is high.

Detailed performance values of the proposed model are presented in Table 1. According to the table, the Precision, Sensitivity, and F1 Score values for the Obstruction class were all 100%. The Normal class had a 100% Sensitivity, while the Flat class had a 100% Precision. This indicates that the model did not produce any false positives in the Flat class predictions and did not miss any cases in the Normal class. The fact that the F1 score is 98.00% or higher for all classes suggests that the model is balanced and successful despite class distribution differences in the dataset.

**Figure 6:**
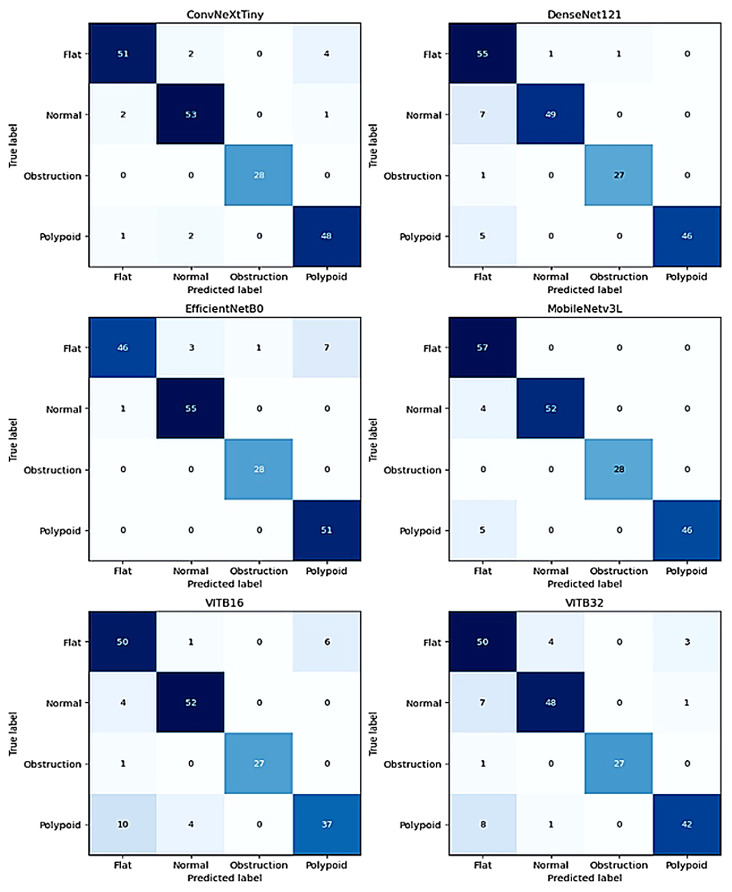
Confusion matrix of pre-trained model.

When examining the complexity matrix of the ViTB32 model used for comparison, as shown in Figure 6, it is observed that the model performs particularly poorly on the Polypoid class. Only 44 out of 51 examples in this class were correctly predicted. 6 examples were confused with the Flat class, and 1 example with the Normal class; furthermore, there were 2 erroneous predictions in the Flat class. Compared to the proposed model, ViTB32 has lower accuracy in class boundary detection.

The results of the ViTB16 model support the idea that ViT-based architectures exhibit relatively lower performance on this dataset. ViTB16 failed in the Polypoid class, correctly classifying 38 out of 51 test images while incorrectly classifying 13. This error rate indicates that the model is unable to distinguish polyp structures from other tissues. Examining the confusion matrix of the EfficientNetB0 model, we see that it performs poorly on the Flat class. While correctly predicting 46 out of 57 Flat class test images, it incorrectly predicted 11. EfficientNetB0 was successful in other classes. DenseNet121 incorrectly predicted 6 test images belonging to the Normal class as Flat. Similarly, it predicted 4 test images belonging to the Polypoid class as Flat. ConvNeXtTiny has a more balanced error rate among the classes. ConvNeXtTiny incorrectly predicted on 12 test images. MobileNetv3L was more successful than ConvNeXtTiny, incorrectly predicting 9 test images. ViTB32 was one of the unsuccessful ViT models, incorrectly predicting 16 of the 16 test images, making it one of the models with the most errors. The proposed model was the most successful, incorrectly classifying 3 test images.

The performance values of all models used in the study and the proposed method are compared in Table 2. The comparison of the table data indicates that the proposed model outperformed all competitors, achieving a test accuracy of 98.44% and a very low error rate of 1.56%. Given the architectural differences, ViT-based models remained in the 86% range, indicating lower performance than CNNs and the proposed hybrid structure. This indicates that the dataset size may be insufficient for non-hybrid structures. Overall, the proposed model was more accurate, precise, sensitive, and had a higher F1 score. It was statistically shown to be a reliable method for classifying sine-wave images.

## 4. Discussion

The morphological assessment of the maxillary sinus membrane is crucial for the success of dental implant surgery and sinus floor elevation procedures. In this study, sinus membrane shapes (Normal, Flat, Polypoid, Obstruction) were classified by specialist physicians in CBCT images. The performance of a unique hybrid DL model based on CNN and ViT and developed for automatic classification was evaluated. According to the findings, the proposed model showed 98.44% test accuracy. Among the models used other than our proposed model, the best results were obtained with ResNet50 (97.92%) and MobileNetV3L (95.31%).

Wu et al. [15], who reviewed DL-based sinus diagnosis studies in the literature, noted that model performance varies depending on the diversity of the dataset, the imaging method used, and the architecture, but generally provides high diagnostic accuracy. The review includes 14 studies that used at least one DL model for detection, segmentation, and classification using panoramic radiography and CBCT. These studies reported success rates ranging from 75.7% to 97.7%. The lowest and highest success rates in the review were reported by Serindere et al. [20] for a CNN-based model for sinusitis detection using panoramic radiography and CBCT images. Wu et al. reported that the highest detection and classification success was achieved by Ha et al. with 92% [15,21]. In their study examining 426 panoramic radiographs, Ha et al. proposed a CNN model to automatically classify retention pseudocysts in maxillary sinuses. Their proposed model achieved 81% detection accuracy for retention pseudocysts. The proposed model achieved 92% overall accuracy for the classification and diagnosis of sinus diseases into three classes (healthy, retention pseudocyst, and cyst or tumor). The 98.44% accuracy achieved in our study places the proposed model among the highest-performing systems in the current literature.

In recent studies, Altun et al. achieved an F1 score of 92.4% for cysts and 88.9% for mucosal thickening using the YOLOv5 architecture, while Öztürk et al. achieved an F1 score of 97% for sinus segmentation using U-Net [22,23]. Hung et al. used the single V-Net model to detect and segment maxillary sinus membrane thickness and retention cysts, noting that the model has significant potential [37]. He et al. stated that the deep CNN-based model provided clinically reliable results in their segmentation study evaluating the maxillary sinus and anatomical structures from CBCT slices [38]. Zeng and colleagues reported a 93% overall accuracy rate with their hybrid model [24]. In a similar study, Alhumaid et al. classified sinus membrane variations as Normal, Opacified, Polyposis, and Retention Cysts using computed tomography (CT) slices and reported an accuracy rate of 95.83% with a hybrid algorithm based on CNN and Swin Transformer [39]. Our proposed hybrid model (CNN-ViT) not only performed segmentation but also achieved F1 scores of 98.25% (Flat), 98.04% (Polypoid), 100% (Obstruction) and 98.44% overal accuracy rate in membrane shape classification, yielding more accurate results than those reported in the literature for morphological classification.

In another study, Murata et al. reported that the detection accuracy for sinusitis remained around 88% in studies conducted on panoramic radiographs [13]. In another study, Serindere et al. conducted the same sinusitis detection study with panoramic radiography and CBCT. The findings showed that CBCT detected sinusitis 20% better than panoramic radiography [20]. Additionally, as seen in the literature, 3D images show a higher accuracy rate of more than 5% compared to 2D images [15]. The use of CBCT sections and the depth of the developed model in our study confirm the diagnostic superiority of three-dimensional cross-sectional analysis compared to two-dimensional imaging.

The most striking clinical finding of the study is the detection of ostium obstruction (“Obstruction” class), a significant risk factor for post-sinus surgery complications, with 100% precision and 100% recall. Shanbhag et al. showed that polypoid lesions and thickenings greater than 5 mm increase the risk of ostium obstruction [10]. The performance of the developed model in this risky class provides the surgeon with a reliable decision-support mechanism to assess the risk of sinusitis in terms of prognosis before sinus-lifting operations. In addition, Phothikhun et al. and Zhang et al. emphasized that sinus mucosal thickenings (Flat) are frequently associated with dental infections and periapical lesions [3,40]. The ability of our model to distinguish between ‘Flat’ and ‘Polypoid’ thickenings with high accuracy allows the clinician to accurately predict the etiology (endodontic/periodontal or rhinological), and Lin et al. can help predict the risk of membrane perforation as indicated by [4]. A comparison of studies in the literature is given in Table 3.

From a clinical perspective, the interpretability of AI models is as vital as their accuracy. Although visual heatmaps were not generated in this study, the proposed hybrid architecture offers intrinsic interpretability through its Self-Attention Mechanism [29]. This component of the ViT allows the model to assign higher attention weights to clinically relevant regions (e.g., the sinus ostium or polypoid projections) while downplaying irrelevant background structures. This mimics the cognitive process of a radiologist focusing on pathological transitions. Additionally, the BiFPN module ensures that decision-making is based on a synthesis of fine boundary details and global anatomical context, reducing the likelihood of decisions based on artifacts. In summary, theoretically, the superior performance of the proposed hybrid model confirms that combining local feature extraction with global context modeling is a more effective strategy for medical image analysis than using single architectures, especially on limited datasets. Practically, this automated classification system acts as a reliable ‘second opinion’ for dentists. It enables the rapid standardization of risk assessment before sinus floor elevation procedures, potentially reducing the incidence of membrane perforations caused by overlooked morphological variations like flat or polypoid thickenings.

### Limitations and Future Perspectives

Despite promising results, this study has several limitations. First, the study was conducted with data from a single center and a single CBCT device. For the generalizability of the model, it needs to be validated on datasets with different voxel sizes and artifacts from various devices. Also, further testing on different datasets and clinical settings is needed. Second, the analysis was performed on 2D cross-sections; future 3D volumetric analysis could further improve diagnostic accuracy. Third, heat maps such as Grad-Cam were not used in this study to make the model more clinically interpretable. It is recommended that these tools be used in future studies to better interpret the model. Fourth, the reliability of AI was not compared to the human factor in this study. Future studies should compare the results of AI with a team consisting of experts, dentists, and students. Until this is done, the responsibility for final diagnosis and surgical planning should always remain with the dentist, and AI should be used to highlight potential risks that might be overlooked. Although the incidence of false positives is low in our model, false positive predictions also require clinician validation to prevent unnecessary treatment changes.

## 5. Conclusions

This study successfully developed and validated a novel hybrid CNN-ViT model for the automated classification of maxillary sinus membrane morphologies on CBCT images. The proposed model outperformed state-of-the-art CNN and ViT architectures, achieving an overall accuracy of 98.44%. Most importantly, the model demonstrated superior diagnostic capability in identifying “Obstruction” and “Polypoid” types, which are critical risk factors for sinus floor elevation procedures. These findings suggest that the proposed hybrid deep learning approach can serve as a reliable, objective, and time-efficient clinical decision support tool for dentists, facilitating safer surgical planning and reducing the risk of postoperative complications.

## Figures and Tables

**Table 1:** Class-based performance metrics of proposed models.

Class	Precision (%)	Recall (%)	F1 Score (%)
Normal	100.00	96.49	98.21
Flat	96.55	100.00	98.25
Polypoid	98.04	98.04	98.04
Obstruction	100.00	100.00	100.00

**Table 2:** Performance metric comparison for all models.

Model	Accuracy Rate (%)	Loss Rate (%)	Weighted Precision (%)	Weighted Recall (%)	Weighted F1 Score (%)
VITB16	86.46	13.54	86.35	86.46	86.31
VITB32	86.98	13.02	87.69	86.98	87.13
DenseNet121	92.19	7.81	93.59	92.19	92.24
EfficientNetB0	93.75	6.25	94.49	93.75	93.68
ConvNeXtTiny	93.75	6.25	93.83	93.75	93.76
MobileNetv3L	95.31	4.69	95.95	95.31	95.37
Proposed Model	98.44	1.56	98.47	98.44	98.44

**Table 3:** Literature review.

Study	Imaging Modality	Task	Model Architecture	Performance
Serindere et al. [20]	Panoramic + CBCT	Sinusitis Detection	CNN-based	75.7–97.7% (range reported in review)
Ha et al. [21]	Panoramic Radiography	Retention Pseudocyst Detection & Classification (3 classes)	CNN	92% acc.
Murata et al. [13]	Panoramic Radiography	Sinusitis Detection	CNN-based	88% acc.
Zeng et al. [24]	-	Sinus Disease Classification	Hybrid DL model	93% acc.
Altun et al. [22]	-	Cyst & Mucosal Thickening Detection	YOLOv5	F1: 92.4%
Öztürk et al. [23]	CBCT	Sinus Segmentation	U-Net	F1 score: 97%
Alhumaid et al. [39]	CT	Membrane Variation Classification (4 classes)	CNN + Swin Transformer	95.83% acc.
Proposed Model	CBCT	Membrane Shape Classification (Normal, Flat, Polypoid, Obstruction)	Hybrid CNN-ViT	98.44% acc.

## Data Availability

The original contributions presented in this study are included in the article. Further inquiries can be directed to the corresponding author.

## References

[1] Rancitelli D., Borgonovo A.E., Cicciù M., Re D., Rizza F., Frigo A.C., Maiorana C. (2015). Maxillary sinus septa and anatomic correlation with the Schneiderian membrane. J. Craniofac. Surg..

[2] Martu C., Martu M.A., Maftei G.A., Diaconu-Popa D.A., Radulescu L. (2022). Odontogenic sinusitis: From diagnosis to treatment possibilities—A narrative review of recent data. Diagnostics.

[3] Zhang L., Zhang Y., Xu Q., Shu J., Xu B., Liu L., Chen H., Hu Y., Li Y., Song L. (2023). Increased risks of maxillary sinus mucosal thickening in Chinese patients with periapical lesions. Heliyon.

[4] Lin Y.-H., Yang Y.-C., Wen S.-C., Wang H.-L. (2016). The influence of sinus membrane thickness upon membrane perforation during lateral window sinus augmentation. Clin. Oral Implant. Res..

[5] Gracco A., Parenti S.I., Ioele C., Bonetti G.A., Stellini E. (2012). Prevalence of incidental maxillary sinus findings in Italian orthodontic patients: A retrospective cone-beam computed tomography study. Korean J. Orthod..

[6] Drumond J.P., Allegro B.B., Novo N.F., de Miranda S.L., Sendyk W.R. (2017). Evaluation of the prevalence of maxillary sinuses abnormalities through spiral computed tomography (CT). Int. Arch. Otorhinolaryngol..

[7] Dogan M.E., Uluısık N., Yuvarlakbaş S.D. (2024). Retrospective analysis of pathological changes in the maxillary sinus with CBCT. Sci. Rep..

[8] Insua A., Monje A., Chan H.-L., Wang H.-L. (2018). Association of inflammatory status and maxillary sinus Schneiderian membrane thickness. Clin. Oral Investig..

[9] Tavelli L., Borgonovo A.E., Re D., Maiorana C. (2017). Sinus presurgical evaluation: A literature review and a new classification proposal. Minerva Stomatol..

[10] Shanbhag S., Karnik P., Shirke P., Shanbhag V. (2014). Cone-beam computed tomographic analysis of sinus membrane thickness, ostium patency, and residual ridge heights in the posterior maxilla: Implications for sinus floor elevation. Clin. Oral Implant. Res..

[11] Kim Y., Lee K.J., Sunwoo L., Choi D., Nam C.-M., Cho J., Kim J., Bae Y.J., Yoo R.-E., Choi B.S. (2019). Deep learning in diagnosis of maxillary sinusitis using conventional radiography. Investig. Radiol..

[12] Kalyvas D., Kapsalas A., Paikou S., Tsiklakis K. (2018). Thickness of the Schneiderian membrane and its correlation with anatomical structures and demographic parameters using CBCT tomography: A retrospective study. Int. J. Implant. Dent..

[13] Murata M., Ariji Y., Ohashi Y., Kawai T., Fukuda M., Funakoshi T., Kise Y., Nozawa M., Katsumata A., Fujita H. (2019). Deep-learning classification using convolutional neural network for evaluation of maxillary sinusitis on panoramic radiography. Oral Radiol..

[14] Schiller L.A., Barbu H.M., Iancu S.A., Brad S. (2022). Incidence, size and orientation of maxillary sinus septa—A retrospective clinical study. J. Clin. Med..

[15] Wu Z., Yu X., Chen Y., Chen X., Xu C. (2024). Deep learning in the diagnosis of maxillary sinus diseases: A systematic review. Dentomaxillofac. Radiol..

[16] Choi H., Jeon K.J., Kim Y.H., Ha E.G., Lee C., Han S.S. (2022). Deep learning-based fully automatic segmentation of the maxillary sinus on cone-beam computed tomographic images. Sci. Rep..

[17] Yilmaz S.Y., Misirlioglu M., Adisen M.Z. (2014). A diagnosis of maxillary sinus fracture with cone-beam CT: Case report and literature review. Craniomaxillofac. Trauma Reconstr..

[18] Ravesh M.N., Ameli N., Vich M.L., Lai H. (2025). Automated classification of midpalatal suture maturation using 2D convolutional neural networks on CBCT scans. Front. Dent. Med..

[19] Shujaat S. (2025). Automated Machine Learning in Dentistry: A Narrative Review of Applications, Challenges, and Future Directions. Diagnostics.

[20] Serindere G., Bilgili E., Yesil C., Ozveren N. (2022). Evaluation of maxillary sinusitis from panoramic radiographs and cone-beam computed tomographic images using a convolutional neural network. Imaging Sci. Dent..

[21] Ha E.G., Jeon K.J., Choi H., Lee C., Choi Y.J., Han S.S. (2023). Automatic diagnosis of retention pseudocyst in the maxillary sinus on panoramic radiographs using a convolutional neural network algorithm. Sci. Rep..

[22] Altun O., Ozen D.C., Duman S.B., Dedeoglu N., Bayrakdar I.S., Eser G., Celik O., Sumbullu M.A., Syed A.Z. (2024). Automatic maxillary sinus segmentation and pathology classification on cone-beam computed tomographic images using deep learning. BMC Oral Health.

[23] Ozturk B., Taspinar Y.S., Koklu M., Tassoker M. (2024). Automatic segmentation of the maxillary sinus on cone beam computed tomographic images with U-Net deep learning model. Eur. Arch. Otorhinolaryngol..

[24] Zeng P., Song R., Lin Y., Li H., Chen S., Shi M., Cai G., Gong Z., Huang K., Chen Z. (2023). Abnormal maxillary sinus diagnosing on CBCT images via object detection and “straight-forward” classification deep learning strategy. J. Oral Rehabil..

[25] Szabó V., Orhan K., Dobó-Nagy C., Veres D.S., Manulis D., Ezhov M., Sanders A., Szabó B.T. (2025). Deep learning-based periapical lesion detection on panoramic radiographs. Diagnostics.

[26] Butnaru O.M., Tatarciuc M., Luchian I., Tudorici T., Balcos C., Budala D.G., Sirghe A., Virvescu D.I., Haba D. (2025). AI efficiency in dentistry: Comparing artificial intelligence systems with human practitioners in assessing several periodontal parameters. Medicina.

[27] Ibraheem W.I., Jain S., Ayoub M.N., Namazi M.A., Alfaqih A.I., Aggarwal A., Meshni A.A., Almarghlani A., Alhumaidan A.A. (2025). Assessment of the diagnostic accuracy of artificial intelligence software in identifying common periodontal and restorative dental conditions (marginal bone loss, periapical lesion, crown, restoration, dental caries) in intraoral periapical radiographs. Diagnostics.

[28] Ardila C.M., Pineda-Vélez E., Vivares-Builes A.M. (2025). Artificial intelligence in endodontic education: A systematic review with frequentist and Bayesian meta-analysis of student-based evidence. Dent. J..

[29] Tan M., Pang R., Le Q.V. (2020). EfficientDet: Scalable and efficient object detection. Proceedings of the IEEE/CVF Conference on Computer Vision and Pattern Recognition (CVPR).

[30] Zhu L., Deng Z., Hu X., Fu C.-W., Xu X., Qin J., Heng P.-A. (2018). Bidirectional feature pyramid network with recurrent attention residual modules for shadow detection. Computer Vision—ECCV 2018.

[31] Vaswani A., Shazeer N., Parmar N., Uszkoreit J., Jones L., Gomez A.N., Kaiser Ł., Polosukhin I. (2017). Attention is all you need. Advances in Neural Information Processing Systems (NeurIPS).

[32] Dosovitskiy A., Beyer L., Kolesnikov A., Weissenborn D., Zhai X., Unterthiner T., Dehghani M., Minderer M., Heigold G., Gelly S. (2021). An image is worth 16×16 words: Transformers for image recognition at scale. International Conference on Learning Representations (ICLR).

[33] Huang G., Liu Z., van der Maaten L., Weinberger K.Q. (2017). Densely connected convolutional networks. Proceedings of the IEEE Conference on Computer Vision and Pattern Recognition (CVPR).

[34] Liu Z., Mao H., Wu C.-Y., Feichtenhofer C., Darrell T., Xie S. (2022). A convnet for the 2020s. Proceedings of the IEEE/CVF Conference on Computer Vision and Pattern Recognition (CVPR).

[35] Howard A., Sandler M., Chu G., Chen L.-C., Chen B., Tan M., Wang W., Zhu Y., Pang R., Vasudevan V. (2019). Searching for MobileNetV3. Proceedings of the IEEE/CVF International Conference on Computer Vision (ICCV).

[36] He K., Zhang X., Ren S., Sun J. (2016). Deep residual learning for image recognition. Proceedings of the IEEE Conference on Computer Vision and Pattern Recognition (CVPR).

[37] Hung K.-F., Ai Q.Y.H., King A.D., Bornstein M.M., Wong L.M., Leung Y.Y. (2022). Automatic detection and segmentation of morphological changes of the maxillary sinus mucosa on cone-beam computed tomography images using a three-dimensional convolutional neural network. Clin. Oral Investig..

[38] He J., Sun M., Huo Y., Huang D., Leng S., Zheng Q., Ji X., Jiang L., Liu G., Zhang L. (2025). A platform combining automatic segmentation and automatic measurement of the maxillary sinus and adjacent structures. Clin. Oral Investig..

[39] Alhumaid M., Fayoumi A.G. (2025). Hybrid CNN—Swin transformer model to advance the diagnosis of maxillary sinus abnormalities on CT images using explainable AI. Computers.

[40] Phothikhun S., Suphanantachat S., Chuenchompoonut V., Nisapakultorn K. (2012). Cone-beam computed tomographic evidence of the association between periodontal bone loss and mucosal thickening of the maxillary sinus. J. Periodontol..

